# A Case Report on Hypocalcemic Cardiomyopathy: A Rare Cause of Cardiac Failure

**DOI:** 10.7759/cureus.43653

**Published:** 2023-08-17

**Authors:** Eleesha A Varghese, Tarun Zachariah

**Affiliations:** 1 General Medicine, West Suffolk Hospital, Bury St Edmunds, GBR; 2 Medicine, Leicester, Northamptonshire and Rutland Deanery, Leicester, GBR

**Keywords:** hypocalcemia, hypoparathyroidism, left ventricular dysfunction, heart failure, cardiomyopathy

## Abstract

We discuss a case of a 48-year-old man who presented with symptoms of breathlessness, orthopnea, and paroxysmal nocturnal dyspnea. Echocardiogram showed severe left ventricular dysfunction with an ejection fraction of 20% and a coronary angiogram performed later was normal. He was initiated on standard medical management for heart failure. Further blood tests showed that he had severe hypocalcemia secondary to hypoparathyroidism. After the correction of hypoparathyroidism using calcium supplementation and alfacalcidol, his ejection fraction improved to 59%, and 12 weeks later, all anti-failure medications were stopped. A clinical diagnosis of hypocalcemic cardiomyopathy due to hypoparathyroidism was made.

The literature review reveals only a few reported cases of heart failure as the initial presentation of dilated cardiomyopathy due to underlying hypocalcemia.

## Introduction

Hypocalcemic cardiomyopathy is recognized to be a rare cause of heart failure, and to our knowledge, less than 100 cases have been described so far. Usual findings include dilated cardiomyopathy and reduced left ventricular ejection fraction. Hypocalcemia can be secondary to hypoparathyroidism, which may be in some cases iatrogenic, i.e., post-surgery. Calcium is an essential element for cardiac contractility. Hence, hypocalcemia affects the normal ventricular systolic and diastolic function. It has been reported in infants due to low levels of vitamin D but is rarely found in adults [1]. It is refractory to regular heart failure treatment regimen and warrants correction of underlying hypocalcemia to improve the ventricular function. Prompt recognition of the condition is vital, as it is one of the rare reversible causes of heart failure. Other reversible causes of heart failure include arrhythmogenic cardiomyopathy, peripartum cardiomyopathy, thyroid disease, inflammatory/viral cardiomyopathy, and Takotsubo cardiomyopathy [2].

## Case presentation

A 48-year-old gentleman presented to the accident and emergency department with shortness of breath for four weeks, which worsened in the last 48 hours. The patient gave a history of orthopnea and paroxysmal nocturnal dyspnea, but no chest pain. He had no significant past medical history. Clinical examination revealed blood pressure of 100/60 mmHg, heart rate of 72 bpm, elevated jugular venous pressure, bilateral pitting pedal edema, and basal crepitations on chest auscultation. Electrocardiogram showed sinus rhythm with a prolonged QTc of 570 milliseconds. Chest X-ray showed bilateral pulmonary congestion and no evidence of cardiomegaly. Blood tests showed normal full blood counts and liver and renal function, erythrocyte sedimentation rate of 6, significantly low calcium level of 1.03 mmol/L (2.15-2.6), elevated phosphate level of 2.70 mmol/L (0.9-1.5), troponin I < 0.02 microgram/L, and significantly elevated pro-brain natriuretic peptide level of 1960 picogram/mL (<100).

Transthoracic echocardiographic examination showed dilated left ventricle with severe left ventricular systolic dysfunction with an ejection fraction of 20% and severely impaired left ventricular diastolic function. There was no evidence of valvular heart disease and estimated pulmonary artery pressure was normal.

An inpatient cardiology review was requested, and an urgent coronary angiogram was done, which was reported to be normal. The endocrinology review suggested further investigations (as given in Table 1), which revealed low parathyroid hormone level of <0.5 pmol/L (0.5-5.5), vitamin D of 88 nmol/L (20-110), albumin at 42 g/L (34-48), alkaline phosphatase at 83 IU/L (35-129), and magnesium at 0.99 mmol/L (0.65-1.05). An ultrasound scan of the neck could not identify any of the parathyroid glands.

**Table 1:** Biochemical investigations

	Reference range	On presentation	After 2 weeks
Calcium	2.15-2.6 mmol/L	1.03 mmol/L	2.39 mmol/L
Phosphate	0.9-1.5 mmol/L	2.70 mmol/L	1.07 mmol/L
Parathormone	0.5-5.5 pmol/L	<0.5 pmol/L	
Vitamin D	20-110 nmol/L	88 nmol/L	
Albumin	34-48 g/L	42 g/L	
Alkaline phosphatase	35-129 IU/L	83 IU/L	
Magnesium	0.65-1.05 mmol/L	0.99 mmol/L	

A clinical diagnosis of hypocalcemic cardiomyopathy due to hypoparathyroidism leading to heart failure was made. The patient was transferred to the intensive care unit and along with typical heart failure therapy (intravenous loop diuretic and oral angiotensin-converting enzyme inhibitor), intravenous calcium followed by regular oral calcium supplementation and alfacalcidol was commenced. Over the next two weeks, his calcium normalized to 2.39 mmol/L, and phosphate normalized to 1.07 mmol/L, while he was continuing to take alfacalcidol 2 microgram once a day and Adcal D3 one tablet twice a day.

At the time of discharge four weeks later, all his heart failure therapy was stopped and he was discharged on just alfacalcidol 2 micrograms once a day. Echocardiogram repeated eight weeks later showed ejection fraction had improved to 59% and the patient had fully recovered.

**Figure 1:**
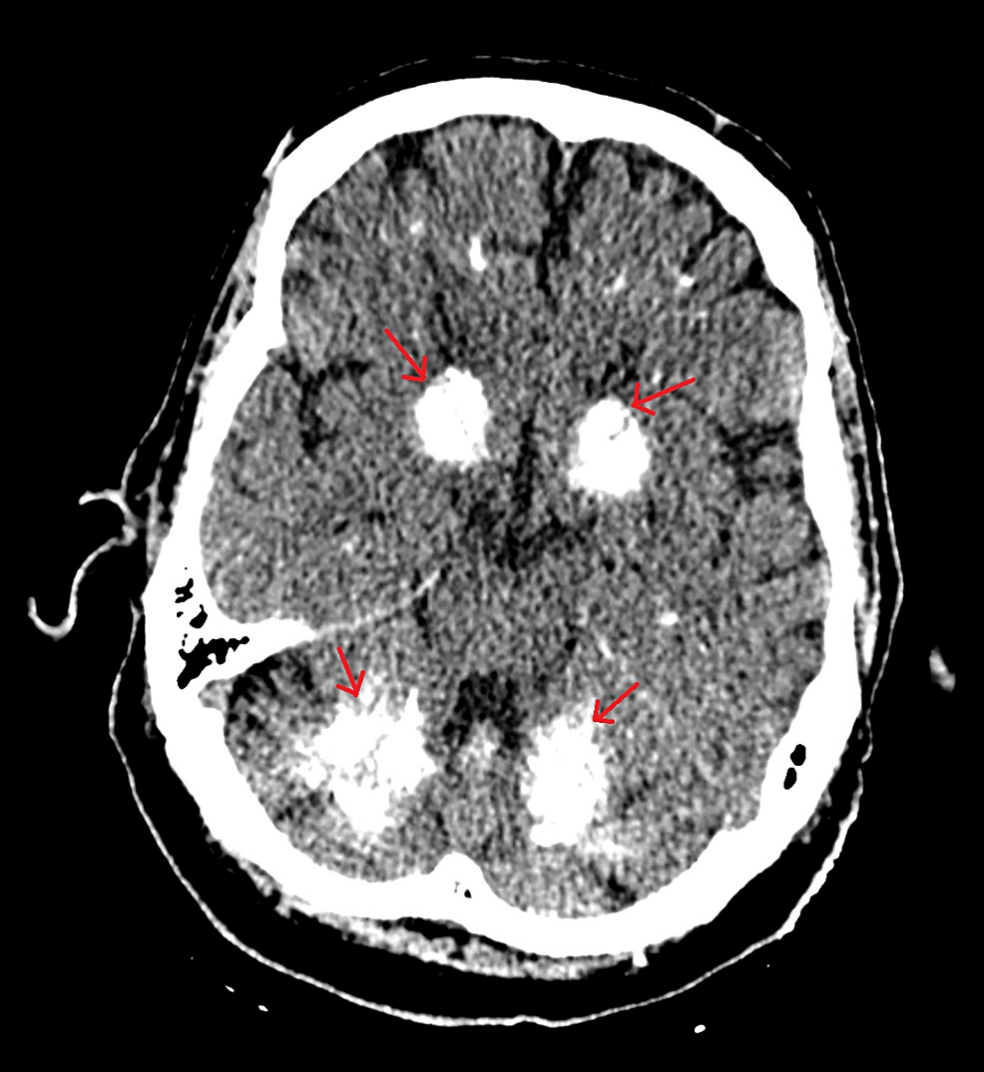
CT of the brain showing bilateral calcifications

Two years later, the patient continued to remain well but presented to the hospital with a possible transient ischemic attack (TIA), and computed tomography (CT) scan of the brain was done, which revealed bilateral symmetrical calcification involving thalami, caudate, lentiform, and dentate nuclei, as well as subcortical white matter (as shown in Figure 1).

## Discussion

This is a rare case of hypocalcemia leading to dilated cardiomyopathy causing heart failure. Hypocalcemia is commonly secondary to hypoparathyroidism, which may be iatrogenic in some cases, i.e., post-surgery. It has been emphasized that the presentations of hypoparathyroidism can vary from asymptomatic cases to an array of symptoms, including serious complications like the above [3].

The clinical presentation depends on the extent and rate of onset of the hypocalcemia. Respiratory manifestations like stridor and laryngospasm, cardiac manifestations like arrhythmias and rarely heart failure, and features of neuromuscular irritability like tetany and seizures are noted in acute hypocalcemia. Ectopic calcium deposition in chronic hypocalcemia can result in extrapyramidal features, dementia, and cognitive impairment [4].

Blood investigations and echocardiogram findings in the above case clearly give a picture of heart failure in the background of hypoparathyroidism. The lack of adequate response to routine heart failure treatment and normal coronary angiogram points toward the diagnosis of hypocalcemic cardiomyopathy. In addition to heart failure, it has also been reported that hypocalcemia can cause arrhythmias. Calcium is important for normal ventricular systolic and diastolic function due to its role in myocardial excitation-contraction coupling. In addition, parathyroid hormone also affects the calcium channels in the myocardium. In some cases, it is noted that hypomagnesemia and vitamin D deficiency can contribute to the worsening of hypocalcemic cardiomyopathy [3].

Intravenous calcium supplementation followed by oral calcium to ensure correction of serum calcium is the mainstay treatment of hypocalcemic cardiomyopathy. Calcium absorption from the intestine is influenced by calcitriol and parathyroid hormone promotes the renal conversion of calcidiol to calcitriol [5]. Hence, it is crucial to treat hypoparathyroidism not only with calcium supplements but simultaneously to prescribe activated vitamin D, as done in the above case. It is also noteworthy to consider the possibility of hypomagnesemia in patients with acute hypocalcemia, which might need correction, as it could be causing reversible hypoparathyroidism [5]. Hence, monitoring and ensuring hemostasis of calcium, phosphate, and magnesium, and parathormone levels are important to avoid complications [6].

Most cases of reported hypocalcemic cardiomyopathy demonstrate significant improvement in left ventricular ejection fraction with normalization of serum calcium. Very few cases document a lack of improvement in the systolic function, which may be attributed to advanced irreversible complications like myocardial fibrosis and degeneration. Since the condition is predominantly reversible with prompt treatment, if recognized and treated appropriately, the prognosis is good. Patient cooperation is vital to ensure good outcomes, as poor compliance can result in the recurrence of the condition.

Some of the notable sequelae of long-term hypocalcemia and hypoparathyroidism are cerebral calcification, cataracts, and cognitive impairment [3]. This is due to metabolic dysfunction in hypoparathyroidism, which causes hyperphosphatemia and consequently elevated serum calcium phosphorous product resulting in ectopic soft tissue calcifications [7]. There have been reported cases of Fahr's syndrome, which is a rare presentation of hypocalcemia due to basal ganglia calcifications [4].

## Conclusions

The clinical presentation of the above case with dyspnea, orthopnea, and pedal edema alongside echocardiographic findings of severe left ventricular dysfunction clearly indicate heart failure. However, refractory cases of heart failure showing inadequate response to routine heart failure treatment should raise the suspicion of the possibility of an underlying metabolic cause, which should be investigated. In this case, the patient was diagnosed to have hypocalcemia secondary to hypoparathyroidism leading to heart failure due to dilated cardiomyopathy. As it is one of the rare and reversible causes, it is crucial to investigate serum calcium and parathyroid levels in unexplained and refractory cases of heart failure. Early recognition and timely management were key in this case. Parathyroid hormone replacement coupled with correction of biochemical disturbances in vitamin D, calcium, phosphate, and magnesium is vital. Long-term treatment may be necessary with good patient compliance to prevent complications, avoid recurrence, and ensure a favorable outcome.

## References

[1] Parepa I, Mazilu L, Suceveanu A, Voinea C, Tica I (2019). Hypocalcemic cardiomyopathy - a rare heart failure etiology in adult. Acta Endocrinol (Buchar).

[2] Patel H, Madanieh R, Kosmas CE, Vatti SK, Vittorio TJ (2015). Reversible cardiomyopathies. Clin Med Insights Cardiol.

[3] Válek M, Roblová L, Raška I Jr, Schaffelhoferová D, Paleček T (2020). Hypocalcaemic cardiomyopathy: a description of two cases and a literature review. ESC Heart Fail.

[4] Mendes EM, Meireles-Brandão L, Meira C, Morais N, Ribeiro C, Guerra D (2018). Primary hypoparathyroidism presenting as basal ganglia calcification secondary to extreme hypocalcemia. Clin Pract.

[5] Bilezikian JP, Brandi ML, Cusano NE (2016). Management of hypoparathyroidism: present and future. J Clin Endocrinol Metab.

[6] Wen Y, Luo X (2022). Hypocalcemic cardiomyopathy: a case report. Front Cardiovasc Med.

[7] Brandi ML, Bilezikian JP, Shoback D (2016). Management of hypoparathyroidism: summary statement and guidelines. J Clin Endocrinol Metab.

